# Surface‐Engineered Cenospheres Encapsulating Phase Change Materials for Functional Cementitious Composites

**DOI:** 10.1002/advs.202417350

**Published:** 2025-04-11

**Authors:** Sahand Rahemipoor, Carsten Kuenzel, Toms Valdemārs Eiduks, Andrei Shishkin, Mohammadreza Izadifar, Neven Ukrainczyk, Eduardus Koenders, Navid Ranjbar

**Affiliations:** ^1^ Department of Civil and Mechanical Engineering Technical University of Denmark Kgs Lyngby 2800 Denmark; ^2^ Department of Civil and Environmental Engineering Imperial College London London SW72AZUK UK; ^3^ Institute of Physics and Materials Science Faculty of Natural Sciences And Technology at Riga Technical University 3 P. Valdena Str. Riga LV‐1048 Latvia; ^4^ Institute of Construction and Building Materials Technical University of Darmstadt 64287 Darmstadt Germany

**Keywords:** cenospheres, etching, interface, molecular dynamics, phase change materials

## Abstract

The escalating global energy demand underscores the critical need for advanced solutions for energy‐efficient buildings. Passive thermal energy storage systems using microencapsulated phase change materials (PCMs) offer promise but face integration challenges in cementitious materials due to weakening mechanical strength, which arises from poor shell strength and weak interfacial bonding with cementitious phases. This study introduces a novel approach for synthesizing functionalized microencapsulated PCMs from fly ash‐based cenospheres addressing interfacial compatibility. Cenospheres are perforated for PCM encapsulation and sealed using two different materials: 1) melamine‐formaldehyde (MF), a standard polymeric shell; and 2) silica, selected for its chemical compatibility with cementitious phases. Experimental results show that the silica sealing improved mechanical strength by 50% over those of MF, corroborated by molecular dynamic simulations showing silica's binding energy with calcium silicate hydrate exceeded threefold, with more than twice the uniaxial tensile strength. Thermal analyses confirmed the preservation of PCM in both sealing approaches. This work establishes a transformative pathway for advancing PCM‐based thermal energy storage in building materials.

## Graphical abstract

The global energy demand drives the need for advanced energy‐efficient building solutions. While current polymeric‐shell microencapsulated PCMs show promise for thermal energy storage, their use in cementitious materials is challenged by poor shell strength and interfacial bonding. This study develops novel cement‐compatible microencapsulated PCM using cenospheres, establishing a transformative pathway for advancing thermal energy storage in building materials.

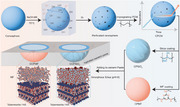

## 1 Introduction

With increasing energy demands driven by population growth and economic expansion, mitigating the 17% contribution of total energy consumption for the heating/cooling system of households has become a critical concern.^[^ ^1^ ^]^ Under these circumstances, passive thermal energy storage, in the central phase change materials (PCMs), offers a promising solution not only to lower energy consumption but also to shift peak loads, reducing energy costs.^[^ ^2^, ^3^ ^]^ PCMs can absorb and release thermal energy during phase transitions within the comfort room temperature range, minimizing indoor temperature fluctuations and enhancing the thermal performance of construction materials.^[^ ^4^, ^5^, ^6^ ^]^ Recent studies have all agreed that micro‐encapsulating PCM is the most visible method for incorporating PCM in building materials amongst other methods including shape stabilizing in porous media and direct incorporation, which addresses issues of liquid‐phase leakage and adaptability present in those approaches.^[^ ^7^, ^8^, ^9^, ^10^, ^11^ ^]^ Currently, polymeric micro‐encapsulation, particularly using melamine‐formaldehyde (MF) via in‐situ polymerization or acrylic polymer via suspension polymerization, is the most common method due to its high encapsulation efficiency, simplicity, and inexpensive fabrication.^[^ ^9^, ^12^ ^]^ Despite the potential, incorporating polymeric micro‐encapsulated PCMs into cementitious composites is not the optimum approach. This is due to a significant reduction in mechanical strength, because of:

‐ Low stiffness and stability of the polymer shell, which is prone to breakage during mixing,^[^ ^13^ ^]^ causing leakage of hydrophobic PCM and hindering cement hydration;^[^ ^14^ ^]^
‐ The poor interfacial bonding/interaction of the microcapsules with hydrate phases in hardened composites can lead to stress cracking and rapid crack propagation.^[^ ^15^ ^]^

To address these weaknesses, attention has recently been put to the possibilities of synthesizing stronger and hydrophilic microcapsules with inorganic shells such as SiO_2,_ CaCO_3,_ and TiO_2_.^[^ ^12^, ^16^ ^]^ Of all, SiO_2_‐based encapsulation has become more appealing in many applications^[^ ^17^, ^18^, ^19^, ^20^ ^]^ due to its simple and controllable synthesis process, superior structural stability, and eco‐friendly nature. However, the current sol‐gel encapsulation method using oil‐in‐water emulsions produces highly disordered structures and is challenged by reproducibility, short shelf life, and leakage during long‐term temperature cycling.^[^ ^12^, ^21^ ^]^ Besides, being more specific to cementitious materials, SiO_2_ offers compatibility and strong interfacial bonding but struggles with durability due to silicate dissolution at high pH, driven by Si─O─Si bond breakage and Si─O^−^ stabilization with alkalis.^[^ ^22^ ^]^ These drawbacks hinder the feasibility of up‐scaling inorganic microencapsulation using these approaches.^[^ ^12^, ^21^ ^]^ Infusing PCM into aluminosilicate hollow microspheres offers a novel alternative. This approach, which involves perforating microspheres, infilling them with PCM, and sealing their surfaces, offers three key advantages: first, compared to polymeric or amorphous inorganic shells, it exhibits superior stiffness and chemical and thermal resistance;^[^ ^23^, ^24^ ^]^ second, it provides flexibility in choosing PCM options such as paraffin wax^[^ ^25^ ^]^ and salt hydrates^[^ ^26^ ^]^; third, its high stiffness allows for a wide range of sealing materials and layers for specific circumstances.^[^ ^26^, ^27^ ^]^

Within the building materials, there is a massive source for hallow microsphere, called cenospheres, which consists ≈1% of coal fly ash, a byproduct of thermal power plants with annual production of 750 million tons.^[^ ^28^, ^29^ ^]^ Cenospheres are obtained in large through hydraulic separation in ponds or using air classifiers. Typically ranging from 20 to 300 µm in diameter and featuring shell thicknesses of 1–18 µm, cenospheres dominantly consist of a strong crystalline scaffold covered by an amorphous aluminosilicate phase. The optimal internal cavity and the distinct alkali‐dissolution rate of the scaffold and amorphous coat, make cenospheres fulfilling major prerequisites for encapsulation applications in construction.^[^ ^23^ ^]^ Key questions are how sealing materials impact the interface between microcapsule and cementitious phases, and to what extent this interaction affects the mechanical properties of cementitious composites.

Herein, we report two surface‐engineered cenospheres with distinct sealing strategies to investigate the critical role of interfacial interactions within the cementitious matrix, examining their impact from the macro to the atomistic scale. For comparison, we selected melamine‐formaldehyde, a widely used polymeric shell in the encapsulation industry, and silica, a nontoxic, cost‐effective, and cement‐compatible material, as sealing options for PCM‐infilled cenospheres. At the initial stage, a comprehensive etching process was performed to perforate cenospheres in an alkaline environment without damaging their spherical geometry, followed by filling with paraffin wax, a PCM with a melting point near room temperature (≈22 °C) to achieve maximum thermal performance for building applications. Our findings reveal that in the same conditions, silica sealing enhances compressive strength by over 50% compared to MF sealing in cement paste, without compromising their thermal properties. Molecular dynamics simulation was performed to study further the intricacies of shell/binder interaction at atomic scales. A basic numerical simulation of a scaled‐up wall indicates a ≈30% reduction in energy consumption under real weather conditions. Our findings point out the significance of interfacial interactions, demonstrating that regardless of shell stiffness, micro‐scale sealing materials profoundly impact mechanical strength by influencing interfacial bonding and interactions between microcapsule surfaces and hydrated phases in cementitious matrixes. These findings present a novel approach to synthesizing cement‐friendly microencapsulated PCM, unlocking the potential for construction materials with superior thermal regulation.

## 2 Results and Discussion

### 2.1 Etching Cenospheres

**Figure 1:**
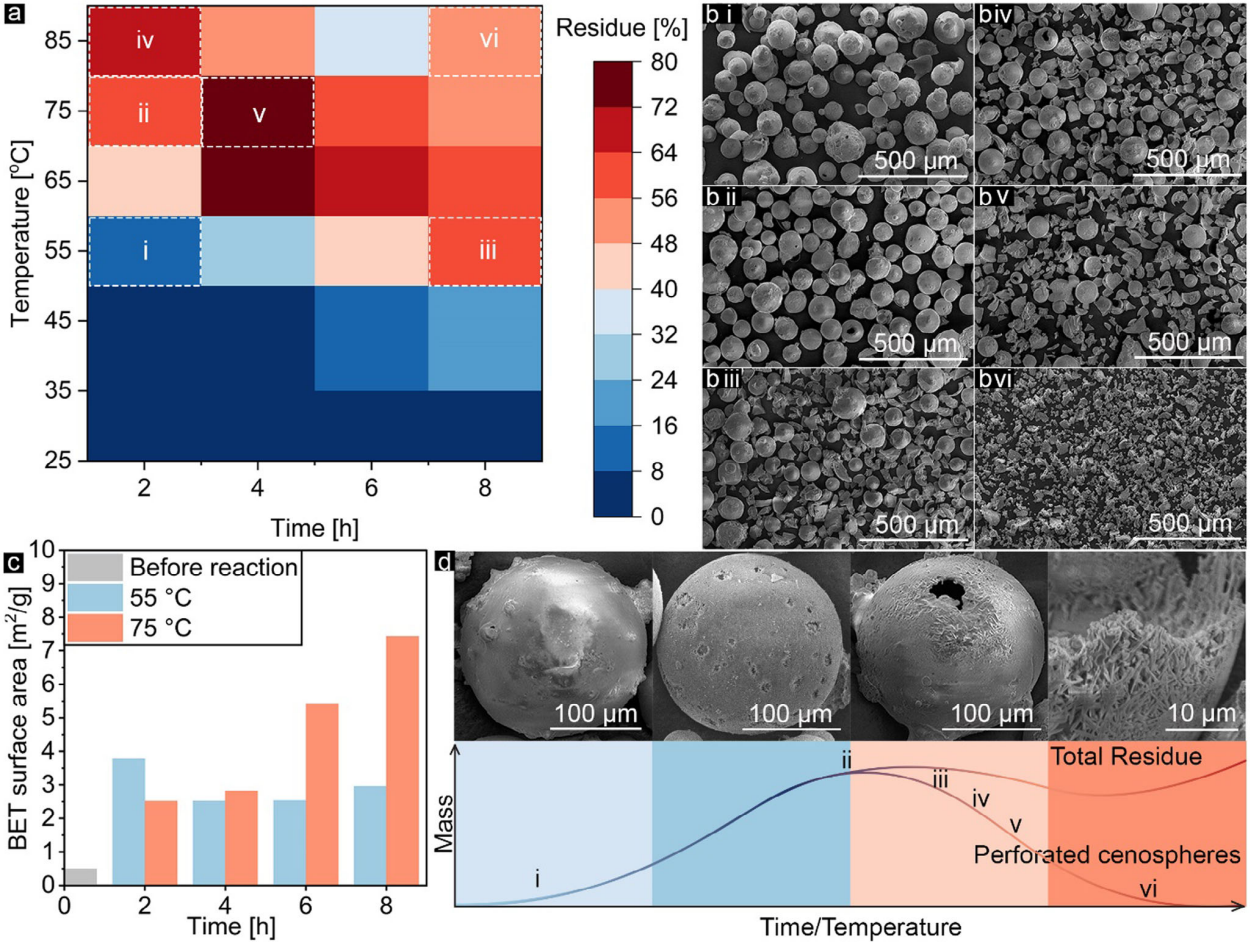
Mass residue results of cenospheres after reaction with NaOH 8M a), SEM images of cenospheres at specified temperature‐time (i–vi indicated in (a)) of reaction b), BET surface area of cenospheres at 55 °C and 75 °C at different reaction duration c), cenospheres etching stage as a function of temperature‐time of reaction d).

Aluminosilicate cenospheres often have a hard (5–7 Mohs scale), compact, impermeable structure that inhibits liquid‐phase ingress into their internal voids.^[^ ^30^, ^31^ ^]^ This structural stability is due to a perforated crystalline scaffold primarily composed of mullite and cristobalite‐quartz, which is covered by an amorphous glassy phase (≈90 wt.%), with minor phases such as gypsum and portlandite also dispersed.^[^ ^23^ ^]^ To make this impermeable surface permeable, the aluminosilicate glassy phase of the cenospheres must be partially and gradually removed through an etching process.^[^ ^32^, ^33^ ^]^ This can be achieved in a highly alkaline solution, breaking covalent bonds and creating perforations on the cenosphere surface.^[^ ^34^ ^]^ Previous studies on the alkaline dissolution of other aluminosilicate materials indicate that dissolution kinetics are strongly influenced by processing time and temperature, while NaOH molarity has a minimal effect beyond concentrations of 8M.^[^ ^32^, ^33^ ^]^ Accordingly, the etching process for cenospheres was examined as a function of time and temperature in 8M NaOH, see **Figure** **1**a. Since cenospheres have a lower density than water and alkaline solutions, the resulting residue comprises perforated cenospheres, fragmented cenosphere structures, and precipitated phases formed during the reaction with NaOH. Based on the results and SEM images (Figure 1b,d), the etching products can be categorized into four stages:

In the first stage, at low temperatures, the cenospheres’ surfaces remain largely smooth, like as received cenospheres. During this stage, a small fraction of cenospheres with structural weaknesses or higher solubility may sink, as structural variation exists among cenospheres.^[^ ^23^ ^]^ Up to 55 °C, due to the negligible dissolution of glassy phases, the residue volume remains low but increases with extended reaction time, mostly due to cenosphere breakage from collisions. This trend is also reflected in the similar BET surface area results observed at 55 °C for varying reaction times, see Figure 1c and Table S2 (Supporting Information). As shown in Figure 1b‐i,iii, it can be observed that while most of the cenospheres kept their spherical geometry up to 2 h of reaction, most were broken after 8 h.^[^ ^3^ ^]^ In the second stage, increasing the temperature up to 75 °C with a reaction time of 2 h led to a significant increase in the residue primarily consisting of perforated cenospheres, as shown in Figure 1a,b‐i,ii. According to Figure 1c, the surface area due to the removal of the smooth glassy surface of the cenospheres, initially 0.5 m^2^ g^−1^, increased to 2,5‐4m^2^ g^−1^ within 2 h, at 55 and 75 °C, respectively. In the third stage, as more of the amorphous phase dissolves and the surface etches further at higher temperatures or longer reaction times, the cenospheres begin to break and lose structural stability, see Figure 1b‐iv,v. For instance, at 65 and 75 °C after a 4‐h reaction, the residue reached the pinnacle, comprising both perforated and broken cenospheres. At 85 °C, the reaction proceeds quickly enough to result in broken cenospheres even after 2 h. With the continued dissolution of the glassy phase at these temperatures, the residue weight decreases after a 6‐h reaction, see Figure 1a. BET surface area results in Figure 1c also demonstrate that the temperature effect is significantly greater than the effect of time due to the faster dissolution of the glassy phase and appearing needle‐like network cenospheres’ scaffold. As more of the glassy phase dissolves, more surface area and pores are created.^[^ ^35^ ^]^ In the final stage, occurring at the highest temperatures and/or longest reaction times, cenospheres lose their structural integrity completely due to the significant dissolution of the amorphous phase and frequent particle collisions, as seen in Figure 1b‐vi which at 85 °C with an 8‐h reaction, the cenospheres' structure is completely destroyed. Additionally, under these conditions, the residue quantity increases compared to the 6‐h reaction, likely due to the precipitation of phases such as aluminosilicate and calcium hydroxide. The etching process is schematically illustrated in Figure 1d.

**Figure 2:**
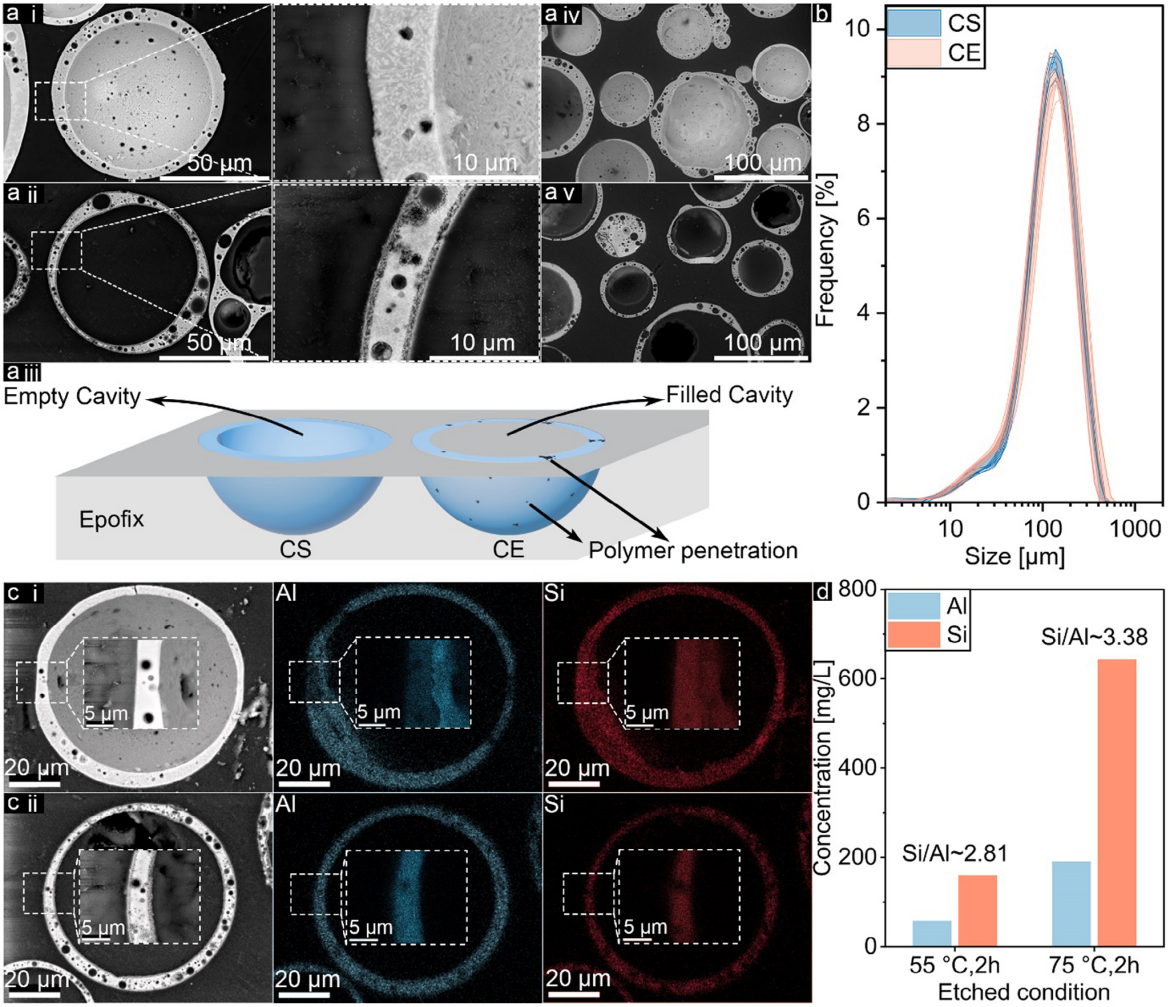
SEM‐BSE images of embedded CS (1,4) and CE (2,5) in epoxy resin and their schematic description (3) a), PSD envelope based on eight measurements for pure cenospheres (CS) and etched cenospheres (CE) in optimum reaction conditions (75 °C and 2 h) b), EDX‐mapping images of CS 1) and CE 2) c), ICP results of CS and CE in different temperature reaction d).

The optimum conditions for the etching process were found at 75 °C and 2 h of reaction by ≈60% residue, mostly perforated cenospheres without damaging their spherical geometry. To gain further insight into the changes in the cenospheres' shells, cross‐sectional SEM images of raw cenospheres (CS) and etched cenospheres (CE), embedded in Epofix resin were captured, see **Figure** **2**a‐i,ii. As evidenced, the surfaces of the CE's wall became more hollow and less condensed compared to the dense wall structure in CS. Additionally, both the interior and exterior surfaces of the cenosphere walls showed visible perforation which indicates that the alkali solution penetrated the internal cavity of the cenospheres. Interestingly, unlike CS which has an empty cavity inside the shell and the entire cenosphere structure is visible, the pores on the CE surface allow Epofix to penetrate the cavity during particle embedding in the polymer under vacuum, as illustrated in Figure 2a‐iii. This phenomenon can be seen for almost all particles as shown in lower magnification in Figure 2‐iv,v. Notably, a small number of CS particles were also filled with polymer which is due to the vacuum pressure during SEM sample presentation that cracked weak particles.

Figure 2b shows the particle size distribution (PSD) for CS and CE. The results indicate no significant difference in size, suggesting that the thickness changes due to etching are likely below the sensitivity threshold of the PSD method. Additionally, these results imply most CE particles hold their spherical geometry, as a negligible increase was observed in the fine particle size range. However, as expected, qualitative energy dispersive spectroscopy (EDS) of the shells showed compositional changes in Al and Si concentrations before and after the etching process. As shown in Figure 2c, unlike in CS, the density of Al became higher than Si in CE. These results align with ICP results from solutions, which showed that the dissolution of Si was more than three times that of Al, resulting in a lower Si content in the CE wall, see Figure 2d and Table S3 (Supporting information). Moreover, the results show that aluminate and silicate do not show the same dissolution kinetics since the Si/Al mass ratio increases with increasing temperature and reaction time. This can be explained by the faster dissolution of aluminate than silicate and the greater effect of temperature on silicate dissolution.^[^ ^32^, ^33^ ^]^ Finally, it should be noted that in CS, the distribution of Si was homogeneous across the wall, but after the etching process, the reduced density of Si may lead to the forming of connected pores and perforated cenospheres.

### 2.2 Functionalization of CE; Filling and Sealing Process

**Figure 3:**
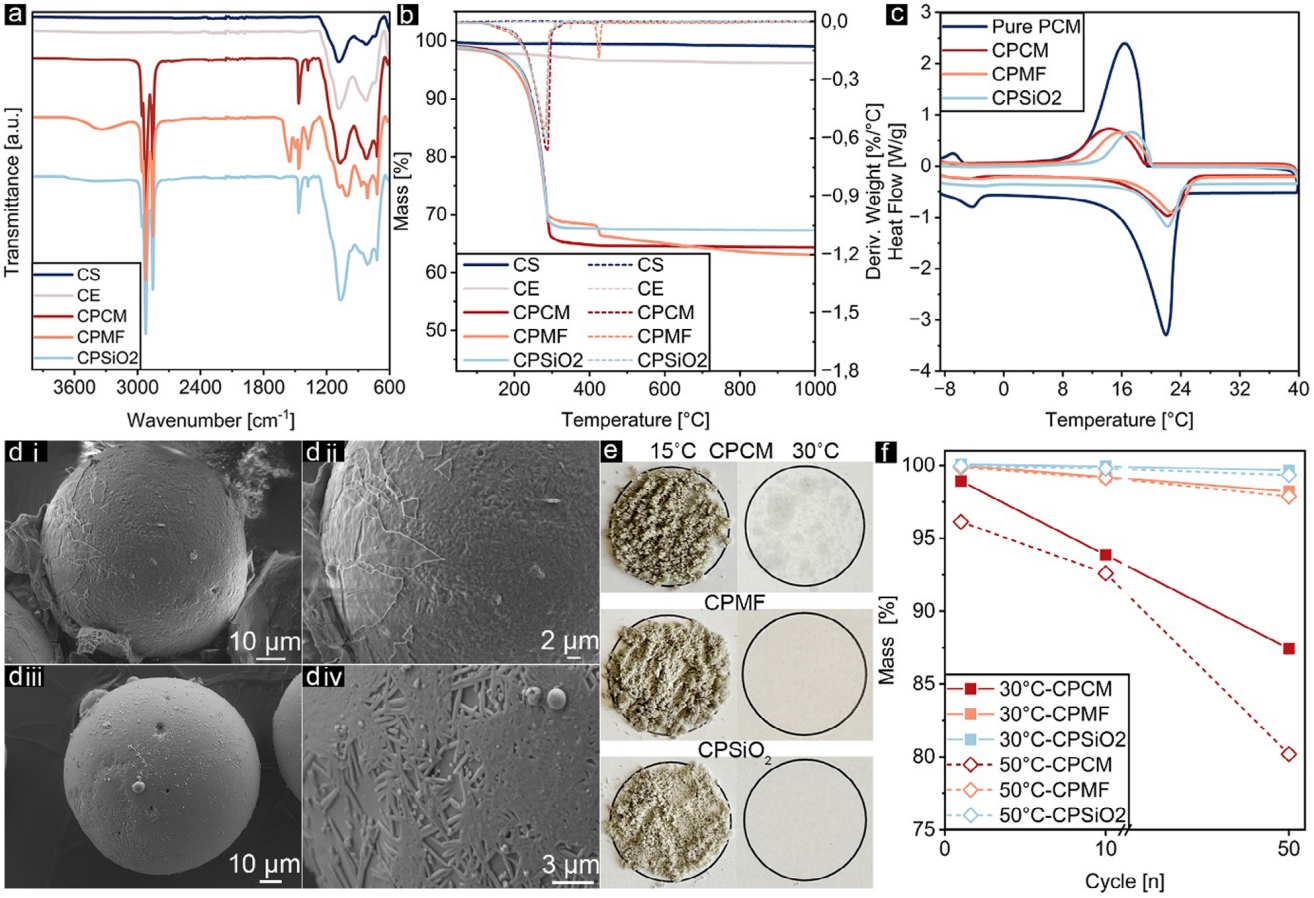
FT‐IR results of all types of cenospheres a), TGA and DTG results of all types of cenospheres b), DSC results of different functionalized cenospheres containing PCM c), SEM images of CPMF (CPCM sealed with MF polymer) i, ii) and CPSiO_2_ (CPCM sealed with SiO_2_) iii, iv) d), and leakage test results of CPCM, CPMF, and CPSiO_2_ at 15 °C (below melting point) and 30 °C (above melting point) on the cellulose paper e), results of the leakage test after multiple cycling of phase changes at 15–30 °C and 15–50 °C f).

A two‐step process was used to functionalize etched cenospheres. In *Step 1*, a vacuum impregnation method was used to infuse PCM (paraffin wax) into the CE cavities (CPCM). In *Step 2*, the infused CE was sealed with two different materials with distinct hydrophilicity behaviors: melamine‐formaldehyde polymer and silica. Stepwise characterization using FT‐IR, DSC, and TGA were employed to quantify the efficiency of each functionalization step. Within *Step 1*, as per the FT‐IR results in **Figure** **3**a, characteristic peaks related to the paraffin wax of PCM were added to the CE spectrum. The peaks at 2853, 2922, and 2958 cm^−1^ represent the C─H stretching vibrations in paraffin wax, while the peaks at 1468, 1379, and 720 cm^−1^ correspond to C─H bending, deformation, and rocking vibrations, respectively.^[^ ^36^ ^]^ In the spectrums of CS, CE, and CPCM, peaks at 1078 and 734 cm^−1^ belong to the stretching vibrations of Si─O─Si and Si─O─Al, as well as the broad peak at 820 cm^−1^ corresponds to both Al─O and Si─O bending vibrations, indicating that the infusing process does not alter the chemistry of the wall.^[^ ^26^, ^37^ ^]^ In TGA results shown in Figure 3b, no significant decomposition or peak was observed for CS, with only a minor 0.9% mass loss likely due to moisture absorption. For CE, overall weight loss was higher at 3.8% which is attributed to the increased surface area and porosity of CE, facilitating greater moisture and water absorption compared to CS. In the DTG results for CPCM, the additional large peak at ≈280 °C corresponds to the decomposition of paraffin wax (PCM), which is not observed in CE and CS. To quantitatively characterize the PCM within the functionalized CE, DSC analysis was conducted, with results shown in Figure 3c and summarized in Table S4 (Supporting information). Using the calculation method outlined in Equation S1 (Supporting Information), over 75% of the CE's void was filled with PCM through this impregnation process. It should be noted that completely filling the void within the cenospheres is theoretically unachievable due to the density differences of PCM in its liquid (0.7 g mL^−1^) and solid (0.76 g mL^−1^) states. In the TGA results for CPCM shown in Figure 3b, the wt.% of PCM in CPCM was calculated as ≈31.8% by comparing the weight loss of CPCM with CE, closely aligning with the DSC result of 35.9 ± 1.0%. Minor differences may be due to the heterogeneous nature of the material.

Despite CE particles being almost completely filled with PCM, the perforated structure of the shells caused the paraffin wax to leach out when it transitioned to liquid at ≈22 °C. Within *Step 2*, two sealing strategies were applied: in situ polymerization of melamine‐formaldehyde (MF) prepolymer to form a flexible MF sealing, and a tetraethyl orthosilicate (TEOS)‐based silica sealing using the Stöber method. The process temperature was kept at 10 °C, below the PCM melting point, to leak prevention. As shown in Figure 3d, the MF forms a thin, flexible crust that covers the micro‐ and sub‐micropores, while the TEOS generates needle‐like crystalline growth on the CPCM surface, effectively sealing cenospheres’ surface by covering the surface pores.

As observed in Figure 3a, the CPMF (CPCM sealed with MF polymer) FT‐IR spectrum illustrates all characteristic peaks of CPCM in addition to the absorbance peaks of MF sealing. These include the narrow peak at 812 cm^−1^ attributed to the out‐of‐plane triazine ring vibration, peaks at 1493 and 1552 cm^−1^ correlated with the in‐plane triazine ring vibration, and a broad peak at 3341 cm^−1^ assigned to N‐H stretching vibration.^[^ ^38^ ^]^ As for CPSiO_2_ (CPCM sealed with SiO_2_), characteristic peaks belonging to CPCM are observed, along with a shoulder peak at 965 cm^−1^ attributed to the stretching vibration of the Si‐OH group in the silica sealing. Furthermore, the intensity of the broad peak at ≈1070 cm^−1^ became more than double compared to CPCM due to the stretching vibrations of Si─O─Si in the added silica sealing.^[^ ^39^ ^]^ According to TGA results for CPMF in Figure 3b, a smaller peak at ≈425 °C was seen, which is related to the decomposition of the melamine‐formaldehyde polymer. Also, the weight loss before the decomposition of melamine‐formaldehyde at 400 °C can be attributed to PCM, as no significant weight loss is observed in CPCM and CPSiO_2_ after 400 °C. In the case of CPSiO_2_, the smaller weight loss compared to CPCM indicates the presence of a silica sealing, which reduces the wt.% of PCM and decreases weight loss associated with PCM decomposition. Besides TGA, DSC results in Table S4 (Supporting Information) demonstrate that sealing CPCM with silica or MF reduced latent heat due to the added weight, which reduced the wt.% PCM. CPSiO_2_ showed a slightly lower latent heat than CPMF which is attributed to silica's higher density, but this difference is negligible. Both CPMF and CPSiO_2_ melted and solidified close to comfortable room temperature, confirming the choice of PCM. For quantitative characterization by TGA, the wt.% PCM in CPSiO_2_ was calculated as 28.9 wt.% by comparing the weight loss of CPSiO_2_ with CE at the end of the test. This result aligns closely with the DSC results (27.0 ± 1.0 wt.%). Based on this analysis, the weight losses attributed to PCM and MF are 27.9 and 5.2 wt.%, respectively. The wt.% PCM calculated by TGA (27.9 wt.%) is in close agreement with the DSC result (28.0 ± 1.0 wt.%), demonstrating the reliability of both methods for measuring PCM content in the mixture.

To visually test the liquid leak prevention‐proof phenomenon after synthesizing, CPCM, CPMF, and CPSiO_2_ were placed on cellulose‐based paper and heated from 15 to 30 °C, then kept for 30 min at 30 °C, as shown in Figure 3e. As observed, CPCM exhibited liquid leakage at the bottom of the powder once the melting point of the PCM was exceeded. However, no obvious leakage was observed in either CPMF or CPSiO_2_, showing that the sealing properly covers the surface pores of the cenospheres. Moreover, a quantitative leakage test was conducted to assess multiple cycling of phase changes based on weight loss due to PCM leakage, and also in higher temperatures (50 °C) to cover changes in the internal volume of the PCMs, see Figure 3f. As observed, although CPCM exhibited more than 20% weight loss after 50 cycles at 15–50 °C, the weight changes of CPMF and CPSiO_2_ remained negligible, <2%.

### 2.3 Interfacial Interactions of Functionalized Cenospheres in Cementitious Composites

The functionalized cenospheres were incorporated into a Portland cement paste with a water‐to‐cement ratio of 0.35 to study their behavior in the cementitious composite. This low water‐to‐cement ratio was chosen to mitigate particle/phase separation, considering the significant buoyancy effect due to the density difference (ρ_*CS*_≈0.4–0.7 g cm^−3^ and ρ_*cement*_ = 3.15 g cm^−3^). Details on sample preparation are provided in the Experimental Section. The interfacial interaction of functional cementitious composites was investigated in the following steps. Initially, Ultrasound Pulse Velocity (UPV) testing was conducted to analyze the impact of cenospheres on the early‐stage hardening evolution of the cement paste by temporal monitoring of sound traveling speed alteration. Next, intricate microstructural characterization and compressive strength testing were performed to evaluate the hardened properties of the cementitious composites. Molecular Dynamics (MD) simulations were used to provide atomic‐scale insights into the interfacial interaction of functionalized cenospheres within the matrix.

**Figure 4:**
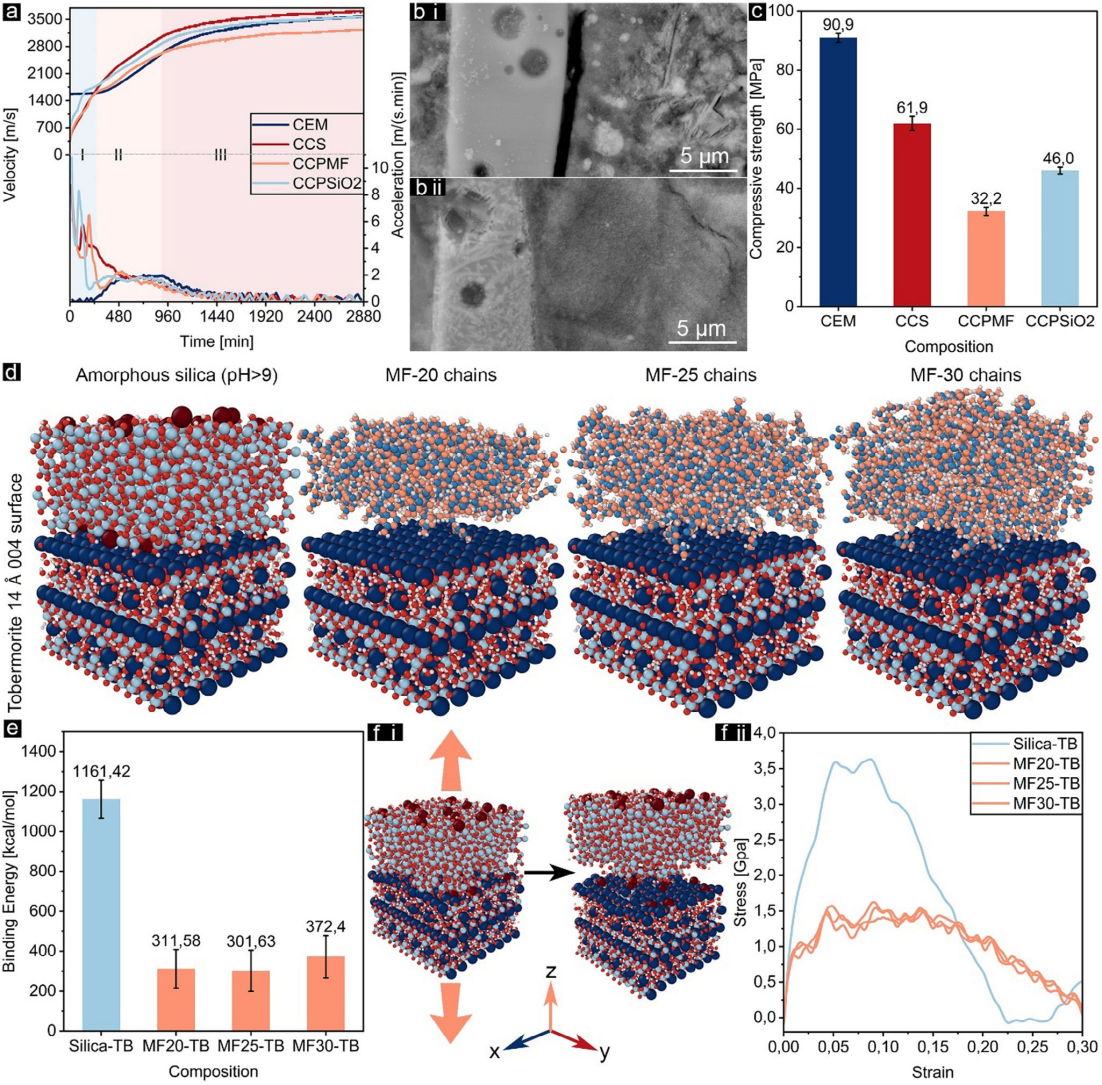
UPV results of fresh pastes without cenospheres (CEM) and with CS (CCS), CPMF (CCPMF), and CPSiO_2_ (CCPSiO_2_) a), SEM images of bonded i) and debonded ii) cenospheres‐cementitious matrix interfaces b), compressive strength of all cementitious composites after 28 days of curing c), virtualization of tobermorite‐14Å (TB)‐silica and tobermorite‐14Å (TB)‐MF with 20, 25 and 30 MF polymer chains d), their binding energy e), and uniaxial tensile simulation in the interfacial (z) direction i) and their results ii) f).

The UPV results for fresh pastes without and with functionalized cenospheres are presented in **Figure** **4**a. The observed UPV evolution for the low water‐to‐binder ratio of cement paste aligns with the three‐stage development pattern: *Stage I*, a dormant stage where the UPV starts at almost constant value (here 1570 ms^−1^) due to trace formation of hydrates; *Stage II*, an acceleration stage where a noticeable increase in UPV is observed as a connected solid network begins to form rapidly; *Stage III*, a deceleration stage where most cement hydrates are fully connected and a solid network is established.^[^ ^40^, ^41^, ^42^ ^]^ In cementitious composites containing cenospheres, the UPV values in *Stage I* were dropped due to increased entrapped air, and the significant difference in sound traveling speed of air (340 ms^−1^) and water (1490 ms^−1^). A similar phenomenon was observed in former studies for cement pastes with higher air content.^[^ ^43^ ^]^ Over time, the formation of hydrate phases is promoted resulting in higher cenosphere‐binder percolation. Since the sound traveling speed of mullite, 3970 ms^−1^,^[^ ^44^ ^]^ is much higher than the cement pastes, the enhanced percolation results in higher UPV value in *Stage II* ^.^ In addition, the interfacial contact between the cementitious phases and cenosphere particles played another key role where strong contact facilitates the transmission of ultrasound. Enhanced hydrophilicity at the shell is favorable to secure the integrity of the composite system since the weak interaction can cause debonding between particles and binders, see Figure 4b. Similar trends continue over time. Noteworthy, the higher UPV values measured for cementitious systems with untreated cenospheres are attributed to the denser structure of the shell compared with the etched ones.

The compressive strengths of cementitious composites with and without functionalized cenospheres are shown in Figure 4c. As expected, the reference cement's compressive strength was reduced by including hollow spheres. However, the properties of the binder‐cenosphere interface significantly affected the composites' strength. The composite that contained silica‐sealed cenosphere showed more than 50% higher strength than those with MF‐sealed particles which can be explained by two mechanisms. First, silicates at the interface can react with calcium hydroxide through a pozzolanic reaction, producing an extra binding phase, calcium silicate hydrates (C─S─H), and providing additional nucleation sites.^[^ ^45^, ^46^ ^]^ Second, the silica has higher hydrophilicity, providing a denser structure at the interfacial transition zone with C─S─H due to the Coulombic interactions of interfacial ions.^[^ ^34^ ^]^ A similar mechanism was responsible for the higher compressive strengths in untreated cenospheres composites due to the much thicker shell, rich in glassy phases. Notable, in those samples the thickness and density of the shell caused them to resist higher loads.^[^ ^47^ ^]^ On the other side, the MF polymer shell induces weaker interfacial interactions within the cementitious matrix due to its softness and higher hydrophobicity than silica. These weak bonds and soft spots in MF‐sealing initiate stress cracks, leading to rapid crack propagation.^[^ ^48^ ^]^ At the atomistic level, Figure 4d illustrates MD simulation models of silica‐tobermorite‐14 Å and MF‐tobermorite‐14 Å with 20 (0.94 g cm^−3^), 25 (1.05 g cm^−3^), and 30 (1.13 g cm^−3^) MF chains. Three different chain numbers were considered to compare the randomness configuration effect on interfacial atoms interacting with the tobermorite‐14 Å. Table S5 (Supporting information) presents the average potential energy of each model during a 1 ns relaxation in four different periods. The results revealed that after 330 ps, potential energy changes were less than 0.5% for all models, confirming that 1 ns was a sufficient duration to reach equilibrium. In addition, the interaction energy and interfacial binding energy of the combined models were calculated using Equation (1):

(1) $$\Delta E=-I.B.E.=E_{system}-\sum E_{Individual}$$

where Δ*E* and I.B.E. represent interaction energy and the interfacial binding energy, *E_system_* and *E_Individual_* refer to the potential energy of the combined model system and each individual model, respectively.^[^ ^49^ ^]^ The comparison of I.B.E. confirmed that silica has a stronger interfacial interaction with tobermorite‐14Å compared to MF, with more than three times higher binding energy, which also aligns with the compressive strength results, see Figure 4e. Furthermore, variations in the binding energy of different MF and tobermorite‐14 Å models confirmed that the number of chains did not have a linear correlation with binding energy. This is because the random placement of chains influences the configuration of atoms at the interface, and a higher number of chains does not necessarily incur greater occupation of the interface due to the randomness and amorphous nature of MF. As shown in Figure 4, uniaxial dynamic tensile test simulations were performed in the interfacial (z) direction to comprehensively understand the mechanical behavior at the interface. As expected, the ductility of MF models was more than the silica model with a longer plastic region. This is possibly due to the greater flexibility and mobility of the MF polymer chains, which can slide past one another, unlike the rigid, fixed structure of silica. Lastly, the peak strength of the silica‐tobermorite‐14 Å model (3.68 GPa) was more than twice that of the MF‐tobermorite‐14 Å models (1.61–1.71 GPa), which is consistent with the binding energy results. This can be attributed to the weak van der Waals and nonbonded interactions between the polymer chains and tobermorite‐14 Å, in contrast to the stronger coulombic interactions between silica and tobermorite‐14 Å surface atoms.

### 2.4 Thermal Performance of Functionalized Cenosphere Incorporated Cement Composites

**Figure 5:**
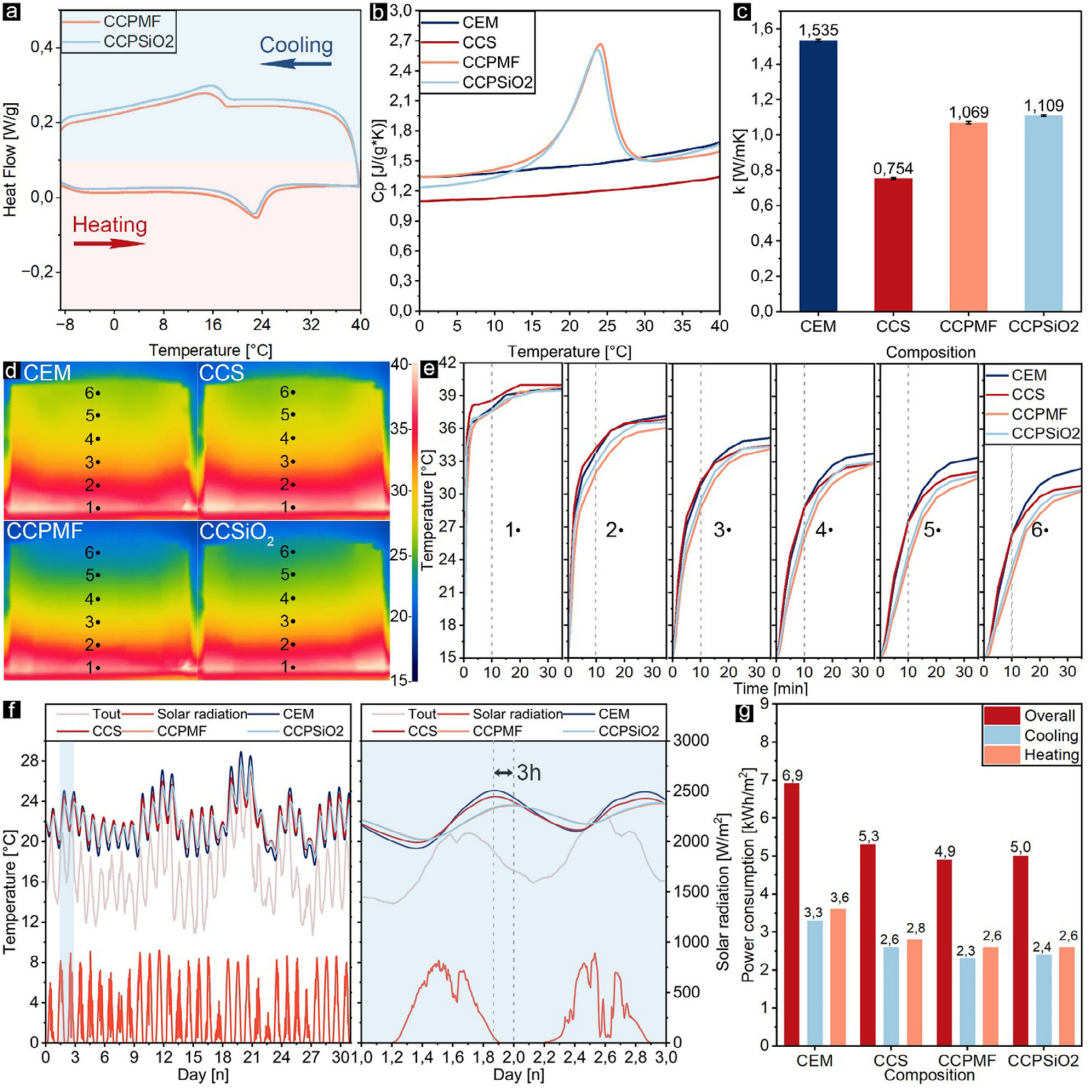
DSC curve of CCPMF and CCPSiO_2_ a), specific heat capacity b) and the thermal conductivity c) of hydrated pastes of all compositions, visual hot‐plate test within 15–40 °C by IR‐camera at 10 min d) and the average temperature of specimens at six different heights e), the outdoor temperature, solar radiation, and calculated interior surface temperatures f), and power consumption g) of simulated walls based on real weather conditions of July in the vicinity of DTU.

Thermal characterization and performance of cementitious composites were compared by measuring latent heat, specific heat capacity, thermal conductivity, thermal performance on the hot plate, and numerical simulations to calculate the power consumption for each composition based on obtained parameters. First, melting‐solidification processes were characterized through a cyclic test and specific heat capacity using DSC analyses, as shown in **Figure** **5**a,b and Table S6 (Supporting information). According to the results, the latent heat obtained from DSC analysis (7.2 ± 0.2 J g^−1^ for functional cenospheres with MF sealing and 6.7 ± 0.4 J g^−1^ for silica sealing) closely matches the calculated latent heat values (7.0 ± 0.3 J g^−1^ and 6.7 ± 0.3 J g^−1^), suggesting that there was no significant PCM loss during the cementitious sample preparation and aging process. Similarly, the specific heat capacity, measured according to ASTM E 1269, showed comparable values for both composites, as illustrated in Figure 5b. Besides, due to the lower thermal conductivity of PCM (≈0.2 W/(m K)),^[^ ^50^ ^]^ both composites exhibited nearly identical, and ≈30% lower thermal conductivity than hydrated cement without cenospheres, as shown in Figure 5c. These results imply that the sealing process did not adversely affect the thermal properties of the functionalized cenospheres. However, comparing samples containing treated and untreated cenospheres showed the significance of infill materials in both specific heat capacity and thermal conductivity.

Considering the heterogeneity in cementitious materials and the limitation of microscale characterization of DSC, a broader evaluation of the bulk thermal performance of the composites was assessed through a thermal diffusivity experiment on a hotplate. An infrared (IR) camera was employed to capture both the temporal and spatial temperature changes during sudden heating from 15 to 40 °C, followed by maintained temperature at 40 °C, see Figure 5d,e, and Figure S4 (Supporting information). At the initial stage of the test in 10 min, the temperature gradient for composites including functional cenospheres was steep near the hotplate due to the dominant role of specific heat capacity and the latent heat of PCM, which suppressed thermal energy diffusion and increased temperature. As a result, by getting distance from the hot plate, the temperature differences between these composites and those without functional cenospheres increased, reaching a peak (3–4 °C) at point 6. Over time and after crossing the specific heat capacity peak of composites including functional cenospheres, their temperatures became closer to the composite with untreated cenospheres, while the hydrated paste without cenospheres showed the highest temperatures due to its higher thermal conductivity. Overall, these results indicate that even with a sharp increase in temperature, composites containing functional cenospheres can effectively slow down temperature changes.

To gain insight into upscaling potentials, a validated 1D reduced‐order finite element model^[^ ^38^ ^]^ was employed to simulate 20 cm‐walls using COMSOL Multiphysics 6.2. For this simulation, actual temperature and solar radiation data from July near the Technical University of Denmark were used; see Figure 5f. Table S7 (Supporting information) presents the assumed and measured parameters applied in the FEM simulations. A general observation of the temperature profile indicates that composites containing functional cenospheres exhibited significantly lower daily temperature fluctuations due mainly to the damping effect of PCM's phase transition. In addition, Power consumption (*P*_consumption_) for a wall made by different composites was calculated using the following Equation (2):

(2)

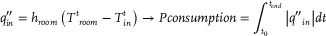

where *q*″_*in*_, *h_room_*,  *T_room_*, *T_in_* represent the heat flux on the interior surface (room) of the wall, the heat transfer coefficient for convection based on the room condition, room temperature which is kept constant at 22 °C using HVAC, and the temperature of the inside surface of the wall. In Figure 5g, both composites included functionalized cenospheres exhibited nearly identical results, showing ≈30% lower energy consumption compared with hydrated paste and ≈6% more than those containing untreated cenospheres. In addition, the temperature profiles for composites including functional cenospheres show significant daily energy consumption shifts up to 3 h, which is important during peak demand times to reduce energy costs (see Figure 5f).^[^ ^51^, ^52^ ^]^ Based on these observations, it can be concluded that the sealing materials did not notably alter the thermal performance of the functionalized cenospheres; however, the mechanical strength was dramatically enhanced when cenospheres with superior interfacial interaction were used.

## 3 Conclusion

In summary, this study introduced a novel approach for synthesizing cement‐compatible microencapsulated PCMs using inorganic‐shell cenospheres, focusing on enhancing interfacial compatibility within cementitious composites. Etching cenospheres was significantly a function of temporal and thermal parameters identified 75 °C and a 2‐h duration as optimal, yielding ≈60 wt.% perforated cenospheres without breaking their spherical geometry. Thermal characterizations confirmed that more than 75% of the cenospheres’ cavities were filled with PCM, and the successful sealing strategies did not affect the thermal performance of the functionalized cenospheres. Early‐stage hardening evolution of cementitious composites revealed that increasing the hydrophilicity of the sealing materials enhanced the integrity of the composite system. The shell/binder interface properties of microencapsulated PCMs significantly influenced the mechanical performance of cementitious composites, which functionalized cenospheres sealed with silica exhibited more than a 50% improvement in compressive strength compared to those sealed with MF polymer, as weak interfacial interactions between particles and binders can incur debonding and reduction in mechanical strength. Molecular Dynamics simulations at the atomistic level verified these findings, showing that silica exhibited more than three times higher binding energy and twice the uniaxial tensile strength with tobermorite‐14 Å compared to MF polymers. Accordingly, MD simulations can be used as a promising approach to pre‐assess the compatibility of surface‐engineered functional materials in cementitious composites. Finally, basic finite element simulations of an up‐scale wall suggested a considerable ≈30% reduction in energy consumption under real‐world weather conditions. These designed approaches and multiscale modeling not only bridge a fundamental gap in PCM‐cement interactions and advance thermal energy regulation in buildings but also pave the way for innovative inorganic shell‐based microcapsules enabling other functions such as self‐healing, fire retardancy, electromagnetic shielding, and lightweight materials.

## 4 Experimental Section

### Raw Materials

Fly ash‐based cenospheres were obtained from coal thermal power plants in Donetsk (Ukraine).^[^ ^53^ ^]^ 2,4,6 Triamino‐1,3,5‐triazine (Melamine), Formaldehyde solution 37%, Acetic acid glacial, and Tetraethyl orthosilicate (TEOS) were obtained from Sigma Aldrich (Germany). The phase change material with the type of RT22HC (melting and solidification range from 20 to 23 °C) was obtained from Rubitherm Technologies GmbH (Germany).^[^ ^50^ ^]^ Aalborg Portland Cement (Denmark) with the specification CEM II/A‐LL 52.5 N (LA) was utilized to prepare the cement pastes.

### Etching Procedure of Cenospheres (CE)

In this study, an 8 m sodium hydroxide (NaOH) solution was used to dissolve the aluminosilicate glassy phase of cenospheres and etch their surface. Previous studies indicated that although the dissolution kinetics of aluminosilicates are highly dependent on temperature and time, NaOH alkaline molarity >8 m does not significantly affect the dissolution rate.^[^ ^33^, ^35^ ^]^ Six different temperatures in four different durations from 2 to 8 h were determined to investigate the optimum condition for the etching process. For each time‐temperature point, 1 *g* of cenospheres was added to 25 mL of 8 m NaOH in a 50 mL Falcon and stirred. After the etching process, the floated particles on top of the solution were first swiped, and then, the sunk particles were separated from the alkaline solution and washed with water to neutral pH. The sunk particles were dried at 105 °C and weighed. The optimum etching parameters were used further for upscaling.

### Filling of CE with PCM (CPCM)

First, 100g of PCM was melted in a beaker at 30 °C to achieve a liquefied PCM. Then, 30 g of CE, with the optimal etching method at 75 °C for 2 h, were added to the beaker. Subsequently, the beaker was placed in a vacuum desiccator for 1 h to remove air from within the cenospheres and maximize the filling of their hollow structures. Finally, the filled particles were filtered and washed with warm water to remove any excess PCM from the particles.

### Sealing CPCM with Melamine‐Formaldehyde Polymer (CPMF)

The melamine‐formaldehyde prepolymer was first prepared by adding 2 g melamine and 8 g formaldehyde to 30 mL distilled water. Then, the pH of the solution was adjusted to pH 9 by adding NaOH solution and heated up to 100 °C for 30 min. The mixture was cooled to room temperature after dissolving all reagents and obtaining the transparent solution. Subsequently, 1.5 g CPCM and 4 mL acetic acid glacial were added to the prepolymer solution and mixed with a vortex at 500 rpm for 1 h. Finally, the solution was filtered and washed several times with distilled water to remove unreacted prepolymers and impurities.

### Sealing CPCM with Silica (CPSiO_2_)

The silica sealing was prepared using the conventional Stöber method. First, 1.5 g of CPCM was added to a solution containing 27.5 mL of ethanol and 10 mL of 12 M NaOH solution. Then, 5 mL of TEOS was added dropwise to the mixture and stirred at 500 rpm for 90 min at room temperature. Finally, the CPSiO_2_ resultant was filtered and washed several times with distilled water.

### Pastes Preparation

All mix designs were prepared with a water‐to‐cement ratio of 0.35. Except for the reference composition (CEM), all other compositions included 20 wt.% of CS, CPMF, or CPSiO_2_ in the solid content (see Table S1, Supporting Information). Initially, cement and cenospheres were mixed for 1 min followed by 2 min of mixing after adding water. The pastes were poured into 2.5 cm cubic molds and cured at 95–100% relative humidity and room temperature for 24 h. After demolding, samples were cured in water under sealed conditions for 28 days.

### Characterization

Malvern 3000 laser diffraction (UK) was deployed to measure cenospheres’ particle size distribution (PSD). Ultrasound pulse velocity (UPV) (Ultra Test GmbH, Germany) was deployed to evaluate the effect of cenospheres at the fresh state of pastes. Measurements were accomplished after 10 min of sample preparation for 48 h at ≈22 °C. The concentration of dissolved ions after the etching process was determined using the ICP technique (Varian 720‐ES). An Attenuated Total Reflectance‐Fourier Transform Infrared (ATR‐FTIR) technique was employed to evaluate the chemical composition and confirm successful synthesis using a PerkinElmer Spectrum 100 FTIR spectrometer (Massachusetts, USA). Measurements were conducted by a frequency range of 4000 to 600 cm^−1^, a resolution of 4 cm^−1^, and an average of eight scans. The thermal conductivity of the hydrated pastes was measured at room temperature using a TCI thermal conductivity analyzer (C‐Therm, Canada) via a modified transient plate source method, following ASTM D7984. The melting and solidification points and the latent heat of cenospheres containing PCM and hydrated pastes containing CPSiO_2_ and CPMF were measured using a TA Instruments DSC Q200 instrument (Delaware, USA). For DSC measurements, all tests were conducted within a temperature range of −10 to 40 °C at a heating rate of 5 °C min^−1^ under a continuous nitrogen gas flow of 50 mL min^−1^. The specific heat capacity of the samples was determined using the ASTM E 1269 standard, sapphire Al_2_O_3_, and NETZSCH DSC 200F3 instrument (Germany). For the quantitative PCM leakage test, a climate chamber (Climacell 111 Evo, MMM Group, Germany) was used to simulate multiple cycles within two temperature profiles of 15–30 °C and 15–50 °C and 50 ± 5% relative humidity. The thermal stability of both functionalized cenospheres and hydrated pastes was assessed using a thermogravimetric analyzer (TGA, NETZSCH STA 449F5, Germany). Initially, all samples were stabilized at 40 °C for 30 min. Then, they were heated from 40 to 1000 °C at a rate of 20 °C min^−1^ under a continuous nitrogen gas flow of 20 mL min^−1^. The microstructure of cenospheres with SE detector was captured using a Quanta FEG 250 Analytical ESEM from Bruker (Massachusetts, USA) with a 5 kV accelerating voltage and a low vacuum chamber with a 100 Pa pressure. To obtain cross‐sections of CS and CE, the samples were cast in SLIP‐LG 100 epoxy resin with S‐HG 140 hardener, following a two‐step process. Initially, the cenospheres were embedded in a small, elongated mold. Struers CitoVac (Denmark) was employed to eliminate excess air bubbles. After allowing the resin to harden for 24 h at room temperature and an additional 4 h at 50 °C, the samples were cut into smaller pieces to increase the examinable area. These smaller sections were then arranged in a mold on their cut faces and encased in epoxy resin once more to have proper polishing. After repeating the hardening process, the samples were sanded and polished using the Struers Tegramin‐20 (Denmark). Then, Backscattered electron (BSE) imaging and EDX measurements (AMETEK Apollo X detector) were performed on the cross‐sections of the cenospheres (CS and CE) as well as the hydrated cement composites. These analyses were conducted using an FEI Nova NanoSEM 650 with a 15 kV accelerating voltage and a working distance of 5 mm. 100kN Criterion C45.105 with a 0.5 mm min^−1^ loading rate was deployed to evaluate the compressive strength of hydrated cement composites. The experimental thermal performance test was conducted using a humidity‐temperature‐controlled chamber along with a hot plate. First, the specimen was mounted on a heater plate inside the chamber. An infrared camera (FLIR T420) was used to monitor the surface temperature of the specimen. The chamber environment was set to a constant 15 °C and 50% relative humidity for 1 h to allow the specimen to reach thermal equilibrium. Once the equilibrium at 15 °C was achieved, the heater was activated, raising the temperature at the bottom of the specimen to 40 °C. These conditions were maintained for 35 min to monitor heat transfer through the specimen.

### Molecular Dynamic (MD) Simulations

This study employed classical molecular dynamics simulations using a large‐scale Atomic/Molecular Massively Parallel Simulator (LAMMPS) to model the evolution of a system of interacting atoms and molecules.^[^ ^54^ ^]^ This method utilizes a set of force fields to describe how atomic and molecular interactions are considered. Among the various classical force fields developed in the literature PCFF, OPLS, and COMPASS, the INTERFACE force fields (IFF) are regarded as the most suitable for describing organic‐inorganic interactions.^[^ ^55^, ^56^ ^]^ The IFF is an extension with validated all‐atom parameters for inorganic compounds, which serves as a plug‐in to materials science‐oriented harmonic force fields like polymer consistent force field (PCFF).^[^ ^56^ ^]^ The equilibrium (004) surface of tobermorite‐14 Å (Ca_5_Si_6_O_16_(OH)_2_.7H_2_O) was taken as a representer of the model C‐S‐H structure.^[^ ^57^, ^58^, ^59^ ^]^ Then a rectangular supercell with dimensions of 45.0592 × 44.55 × 120 Å with ≈92 Å vacuum space in the Z direction was created to eliminate boundary effects on interface direction. Furthermore, like the tobermorite‐14Å model, the amorphous silica at pH > 9 with 0.85 SiO^−^Na^+^ per nm^2^ was sourced from the IFF database. The model was configured with the same size of tobermorite‐14Å dimensions and included a 90 Å vacuum space in the z‐direction.^[^ ^60^ ^]^ Subsequently, to achieve dynamic equilibrium for tobermorite‐14Å and silica models, the canonical ensemble (NVT) was utilized with a constant number of atoms and volume and a Nose‐Hover thermostat to keep the temperature constant at 298 K for 1 ns. The last 10 ps of the MD simulation was considered for analysis. To model the melamine‐formaldehyde polymer (MF), a polymer chain consisting of 10 melamine and 9 formaldehyde monomers (Figure S1a in the Supporting Information) was first prepared using the Xenoview program and PCFF.^[^ ^61^ ^]^ Subsequently, three amorphous models containing 20, 25, and 30 polymer chains were created. Later, these three MF models were conducted to isobaric‐isothermal ensemble (NPT) for 1 ns to obtain stable polymer configuration under room pressure (1atm) and temperature (298 K) conditions. Finally, these MF models were confined with the same size of tobermorite‐14 Å periodic cell (45.0592 × 44.55 × 120 Å) in which each model has between 90 to 95 Å vacuum space in the z‐direction to have the same simulation box as tobermorite‐14Å. NVT ensemble at 298 K for 1 ns was applied to those systems until they reached equilibrium, see Figure S1b–d (Supporting Information). To analyze interfacial interactions, the silica, and three MF models were positioned 2 Å above the tobermorite‐14Å within simulation boxes of 45.0592 × 44.55 × 150 Å, including ≈90 Å of vacuum space. These models were subjected to NVT ensemble at 298 K for 1 ns to reach equilibrium and the last 10 ps of the simulations were used for analysis. Finally, the resultants were used for uniaxial dynamic tensile simulation in the Z direction. Systems were tensioned uniformly at a constant strain rate of 5 × 10^−4^ per fs under NVT ensemble at 298 K with a time step of 1 fs. All systems were visualized using the OVITO application.^[^ ^62^ ^]^

## Conflict of Interest

The authors declare no conflict of interest.

## Supporting information



Supporting Information

## Data Availability

The data that support the findings of this study are available on request from the corresponding author. The data are not publicly available due to privacy or ethical restrictions.
