# Supplementary material for: Surface‐Engineered Cenospheres Encapsulating Phase Change Materials for Functional Cementitious Composites
Source: Adv Sci (Weinh). 2025 Apr 11;12(26):2417350. doi: 10.1002/advs.202417350 (PMC12245073; doi:10.1002/advs.202417350)
Supplement: Supplementary file 1 — Supporting Information [file ADVS-12-2417350-s001.docx]

Supporting Information

Surface-Engineered Cenospheres Encapsulating Phase Change Materials for Functional Cementitious Composites

*Sahand Rahemipoor^a^, Carsten Kuenzel^b^, Toms Valdemārs Eiduks^c^, Andrei Shishkin^c^, Mohammadreza Izadifar^d,^*, Neven Ukrainczyk^d^, Eduardus Koenders^d^, Navid Ranjbar^a,^**

*^a^Department of Civil and Mechanical Engineering, Technical University of Denmark, 2800 Kgs Lyngby, Denmark*

*^b^Department of Civil and Environmental Engineering, Imperial College London, London, UK*

*^c^Institute of Physics and Materials Science, Faculty Of Natural Sciences And Technology at Riga Technical University, 3 P. Valdena Str., Riga, LV-1048, Latvia*

*^d^Institute of Construction and Building Materials, Technical University of Darmstadt, 64287 Darmstadt, Germany*

*Corresponding authors: Mohammadreza Izadifar (izadifar@wib.tu-darmstadt.de), Navid Ranjbar (naran@dtu.dk)

Keywords: Cenospheres, Phase change materials, Molecular dynamics, Etching, Interface.

Mix designs of all pastes are shown in **Table S1.**

**Table S1.** Composition of pastes.

| Composition | Cement (g) | | Cenospheres (g) | CPSiO_2_ (g) | CPMF (g) | Water (g) |
| --- | --- | --- | --- | --- | --- | --- |
| CEM | | 100 | 0 | 0 | 0 | 35 |
| CCS | | 100 | 25 | 0 | 0 | 35 |
| CCPSiO_2_ | | 100 | 0 | 25 | 0 | 35 |
| CCPMF | | 100 | 0 | 0 | 25 | 35 |

Figure S1 displays the structure of a single polymer chain of melamine-formaldehyde (MF) used in the molecular dynamics simulations, as well as images of MF with 20, 25, and 30 chains after relaxation through the NVT ensemble within the same simulation box dimensions of tobermorite (TB).


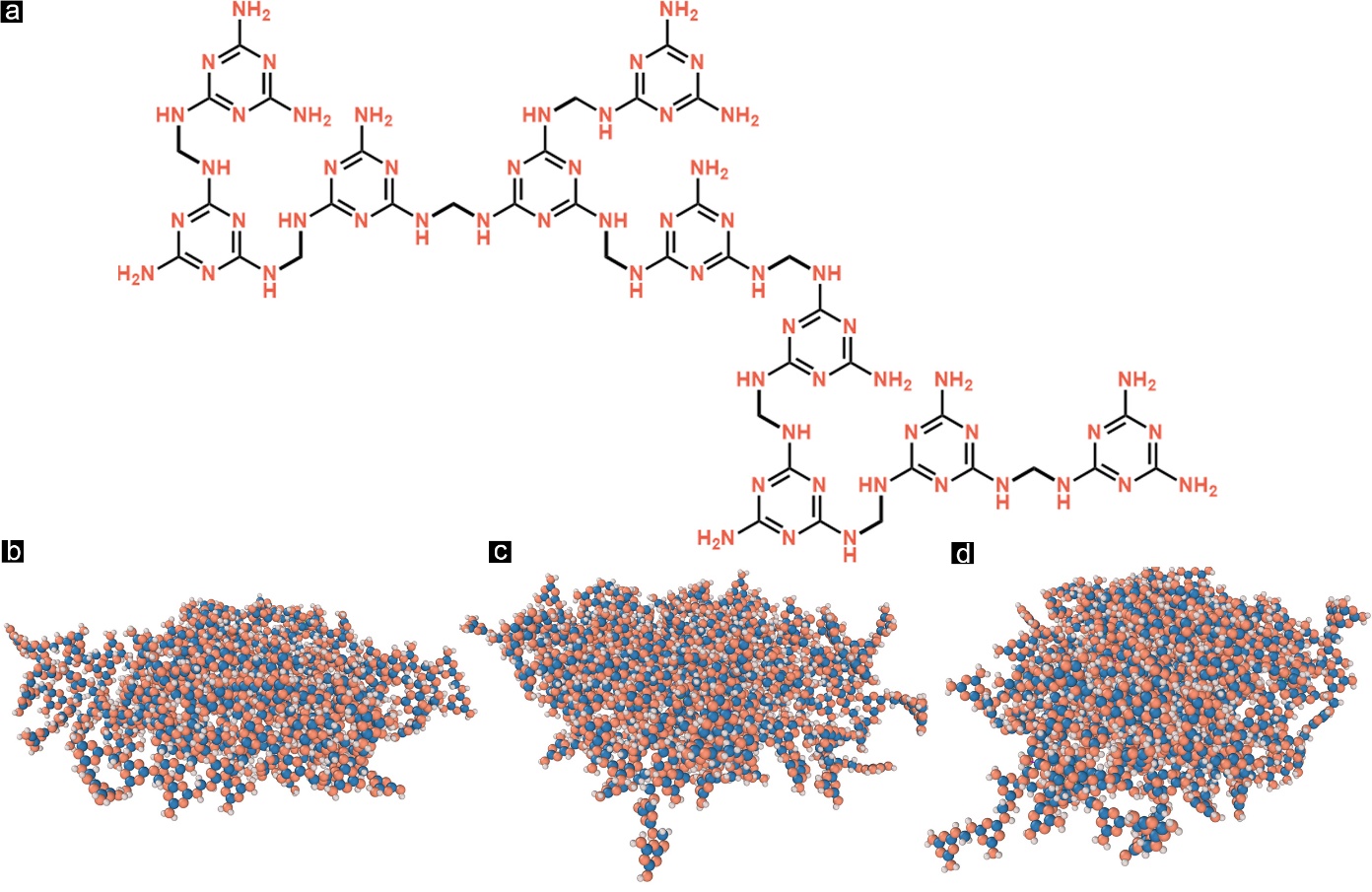


**Figure S1**. Structure of single chain of MF (a) and relaxed 20 (b), 25 (c), and 30 (d) chains of MF.

The BET surface area of floating and sunken cenospheres is provided in **Table S2**. Similar to the trend observed with the sunken particles, for the floating particles an increase in duration of reaction led to increasing in BET surface area. Furthermore, the higher BET values at 75 °C compared to 55 °C can be attributed to the precipitation of aluminosilicate and calcium hydroxide phases.

**Table S2.** BET results of sunken cenospheres after reaction with NaOH 8M [m^2^/g].

| Time [h] | Floating at 75 °C | sunken at 75 °C | Floating at 55 °C | sunken at 55 °C |
| --- | --- | --- | --- | --- |
| 2 | 2.060 | 2.511 | 1.000 | 3.789 |
| 4 | 3.157 | 2.816 | 1.509 | 2.516 |
| 6 | 11.127 | 5.419 | 1.485 | 2.529 |
| 8 | 11.395 | 7.422 | 1.686 | 2.956 |

ICP results from solutions after 2 and 8 hours of reaction in 8M NaOH at temperatures of 55 °C and 75 °C are presented in **Table S3** to follow the compositional changes during the reaction.

Increasing the temperature and the reaction time led to the release of more Al, Si, and Fe ions, which is consistent with the hypothesis of the dissolution of the amorphous glass phase in these conditions. However, the calcium ion concentration decreases over time, most likely due to Ca(OH)_2_ precipitation.

**Table S3.** ICP results of cenospheres solution after reaction with NaOH 8M.

| Sample Labels | Al | Si | Fe | Ca | Si/Al |
| --- | --- | --- | --- | --- | --- |
| 55 °C,2h | 56.5210 | 158.8557 | 9.0205 | 2.9203 | 2.810563 |
| 55 °C,8h | 217.3533 | 738.1944 | 20.2909 | 1.6656 | 3.396288 |
| 75 °C,2h | 190.0458 | 642.8186 | 19.1245 | 1.4821 | 3.38244 |
| 75 °C,8h | 1167.3155 | 4897.6430 | 118.2265 | 0.4081 | 4.195646 |

**Table S4** presents the DSC results for both PCM and functionalized cenospheres, along with the calculated wt. %PCM derived from the latent heat measurements. Since materials other than PCM do not show any peaks within this temperature range, the wt.% PCM in the composite was determined using Eq. S1:

$wt. \%PCM=\frac{{\Delta H}_{functional cenosphere}}{{\Delta H}_{\mathrm{PCM}}}\times100$ (S1)

With an average particle diameter of 130 µm, a 5 µm wall thickness in CE, and density of liquid PCM, the maximum theoretical PCM content in CPCM is calculated to be ~47 wt.%.

**Table S4**. DSC results of RT22HC PCM and different functionalized cenospheres containing PCM.

| Composition | $\boldsymbol{\Delta}\mathbf{H}_{\boldsymbol{m}}$ (J/g) | T_o,m_ (˚C) | T_p,m_ (˚C) | $\boldsymbol{\Delta}\mathbf{H}_{\boldsymbol{f}}$ (J/g) | T_o,f_ (˚C) | T_p,f_ (˚C) | Wt. %PCM |
| --- | --- | --- | --- | --- | --- | --- | --- |
| Pure PCM | 161.8±4.2 | 16.7±0.1 | 22.1±0.1 | 164.5±4.7 | 19.2±0.1 | 16.2±0.2 | 100 |
| CPCM | 57.1±1.7 | 16.4±0.1 | 22.2±0.1 | 58.8±1.8 | 19.0±0.1 | 14.4±0.3 | 35.9±1.0 |
| CPMF | 44.6±1.9 | 17.8±0.1 | 22.9±0.2 | 46.2±1.9 | 19.8±0.1 | 15.6±0.2 | 28.0±1.0 |
| CPSiO_2_ | 42.8±2.1 | 18.1±0.1 | 22.4±0.1 | 44.6±1.9 | 20.0±0.1 | 16.9±0.2 | 27.0±1.0 |

After curing cementitious composites for 28 days and crushed to obtain fine powder, FT-IR spectra were obtained to evaluate the chemical properties of functionalized cenospheres in hydrated pastes, see **Figure S2a**. As expected, all samples exhibited peaks associated with the cementitious matrix, such as the broad peak at 951 cm^-1^ (Si-O stretching in C-S-H), the peak at 3640 cm^-1^ (O-H stretching in Ca(OH)_2_), the peak at 1418 cm^-1^ (C-O asymmetric stretching in CaCO_3_), and the peak at 1110 cm^-1^ (asymmetric stretching of SO_4_^2-^). Considering that more than 80 wt.% of the samples consist of hydrated cement, the peaks related to cenospheres are merged with hydrated cement. As a result, there is no significant difference in the CCS spectrum compared to CEM. However, in the spectra of CCPMF and CCPSiO_2_, peaks at 2853, 2922, and 2958 cm^-1^ (C-H stretching vibration in paraffin) and a shoulder peak at 1468 cm^-1^ (C-H bending vibration in paraffin) were observed. Additionally, in the spectrum of CCPMF, the peak related to MF's in-plane triazine ring vibration appears at 1575 cm^-1^. In the TGA/DTG results of hydrated pastes, as expected, peaks associated with CSH-Ettringite, Portlandite, and Calcite were observed at 135°C, 470°C, and 775°C in **Figure S2b**, respectively ^[15–17]^. In the case of CCPMF and CCPSiO_2_, the decomposition peak of PCM in the cavity of the CE appeared at ~250°C. Since the composition of these pastes including cenospheres is heterogeneous, quantitative analysis of the results was not feasible. However, CCS with 80 wt.% hydrated cement exhibited lower mass loss than CEM, as the cenospheres showed less than 1 wt.% mass loss up to 1000°C. Both CCPSiO_2_ and CCPMF displayed greater mass loss than CCS due to the decomposition of PCM. Also, CCPMF had a larger mass loss than CCPSiO_2_ due to the decomposition of MF in CPMF, as previously discussed. These results indicate that the addition of functionalized cenospheres does not alter the chemical composition of hydrated cement.


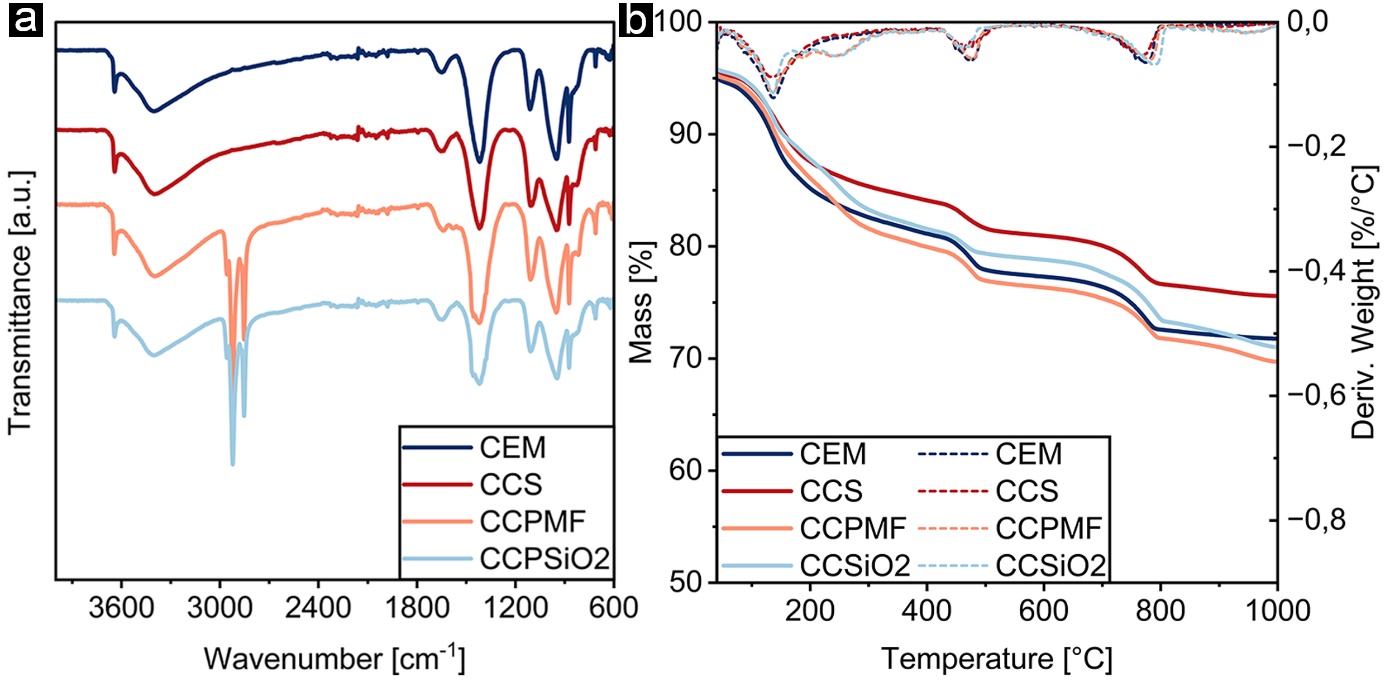


**Figure S2.** FT-IR (a), and TGA-DTG (b) results of cementitious composites.

The cross-sectional optical images of hydrated composites are presented in **Figure S3**, confirming the uniform dispersion of cenospheres within the paste.


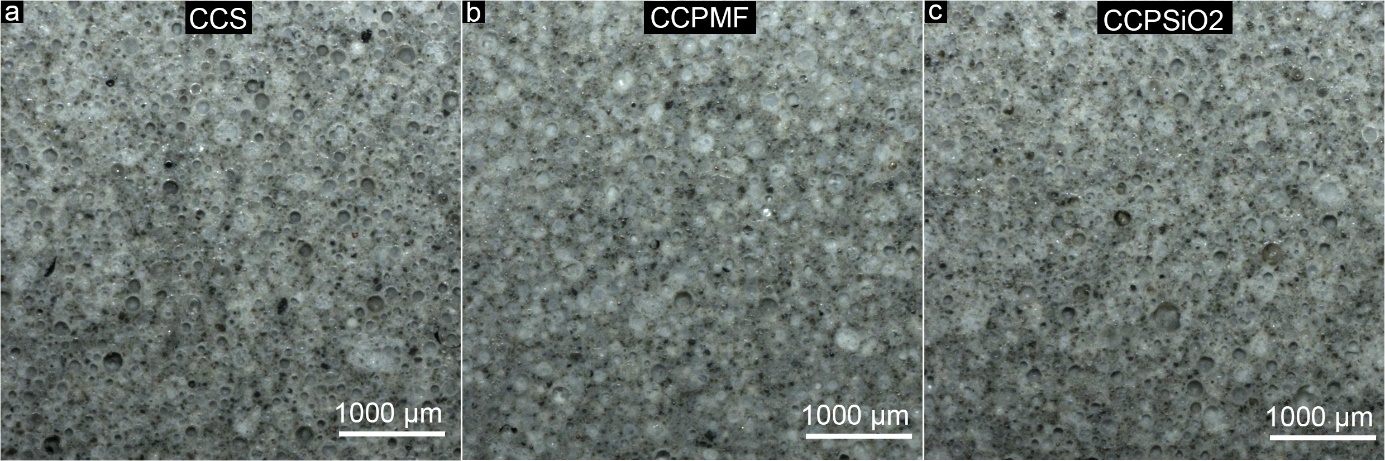


**Figure S3. cross-sectional optical images of hydrated CCS (a), CCPMF (b), and CCPSiO_2_ (c).**

Table S5 summarizes the average potential energy (P.E.) and interaction energy of all models during a 1 ns relaxation in 4 different periods**.**

**Table S5.** Average potential energy and interaction energy of all models. (units: kcal/mol)

| Model | P.E.(0-330 ps) | P.E. (330-660 ps) | P.E. (660-990 ps) | P.E. (last 10 ps) | Interaction energy |
| --- | --- | --- | --- | --- | --- |
| Silica | 293324.37±55.37 | 293323.53±39.71 | 293324.16±39.78 | 293328.49±39.64 | - |
| TB | -53808.75±94.64 | -53820.39±55.96 | -53802.30±56.37 | -53816.49±55.69 | - |
| MF20 | 9771.65±61.71 | 9751.80±44.87 | 9734.70±43.61 | 9759.17±40.79 | - |
| MF25 | 29777.27±74.05 | 29730.51±51.22 | 29707.66±49.78 | 29705.79±48.34 | - |
| MF30 | 45879.48±84.38 | 45852.75±55.15 | 45841.19±53.99 | 45849.73±51.25 | - |
| Silica-TB | 238490.16±113.05 | 238415.56±69.59 | 238376.88±72.49 | 238350.58±66.34 | -1161.42±95.19 |
| MF20-TB | -44364.87±150.83 | -44407.05±70.14 | -44384.35±73.12 | -44368.90±67.54 | -311.58±96.57 |
| MF25-TB | -24351.69±152.57 | -24386.37±73.68 | -24414.23±74.94 | -24412.33±71.86 | -301.63±102.94 |
| MF30-TB | -8243.40±161.38 | -8275.63±77.88 | -8301.89±78.50 | -8339.16±75.12 | -372.40±106.56 |

**Table S6** summarizes the DSC results for CCPMF and CCPSiO_2_ as well as their calculated latent heat values. Since the latent heat is directly related to the mass ratio of PCM in the composite, the melting latent heat of the compositions was calculated based on the melting latent heat of CCPMF and CCPSiO_2_ using Eq. (S2):

$\Delta H_{m,c}=\Delta H_{CPMF or CPSiO_{2}}\times\frac{W_{CPMF or CPSiO_{2}}}{{(W}_{\mathrm{Cement}}{+W}_{\mathrm{Water}}+W_{CPMF or CPSiO_{2}})}$ (S2)

where $\Delta H_{m,c}$ and W represent the calculated latent and the weight of components in the mixture, respectively.

**Table S6.** DSC results for CCPMF and CCPSiO_2_.

| Composition | $\boldsymbol{\Delta}\mathbf{H}_{\boldsymbol{m,c}}$ (J/g) | $\boldsymbol{\Delta}\mathbf{H}_{\boldsymbol{m}}$ (J/g) | T_o,m_ (˚C) | T_p,m_ (˚C) | $\boldsymbol{\Delta}\mathbf{H}_{\boldsymbol{f}}$ (J/g) | T_o,f_ (˚C) | T_p,f_ (˚C) |
| --- | --- | --- | --- | --- | --- | --- | --- |
| CCPMF | 7.0±0.3 | 7.2±0.2 | 15.9±0.5 | 22.0±1.1 | 6.9±0.1 | 18.6±0.3 | 14.5±0.5 |
| CCPSiO_2_ | 6.7±0.3 | 6.7±0.4 | 16.8±0.3 | 22.6±0.2 | 6.4±0.1 | 18.9±0.4 | 15.3±0.5 |

Actual and IR images of specimens used for thermal property evaluation are shown in **Figure S4**.


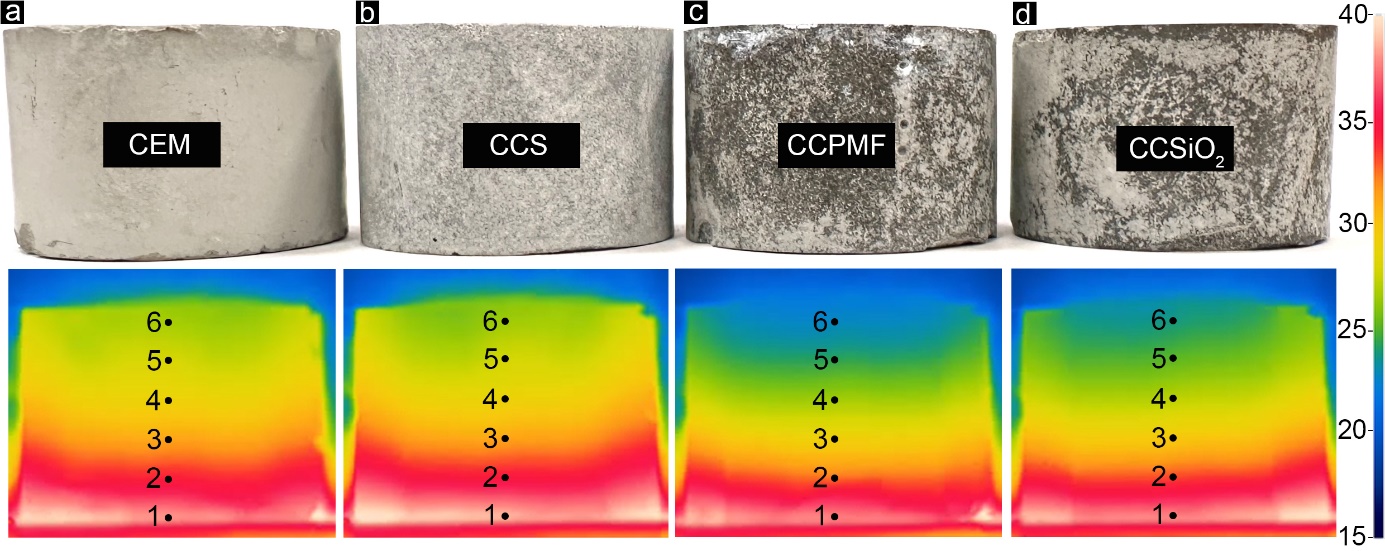


**Figure S4. Actual and IR images of CEM (a), CCS (b), CCPMF (c), and CCPSiO_2_ (d).**

Based on the validated method from our previous study, ROM-FEM simulations were conducted to assess the numerical thermal performance of each composition by calculating the internal surface temperature (T_in_) of walls. **Table S7** lists the parameters considered in the model, which include the following assumptions:

- Composites were assumed to be homogeneous with constant thermal conductivity.
- No internal heat generation from composites, occupants, or devices was considered.
- Convection effects in melted PCM and radiative cooling of the indoor environment were disregarded.
- The left boundary condition was set to convection with a heat transfer coefficient $h_{out}=$25 W/(m^2^·K) and exposed to actual temperature and solar radiation data provided by Meteomatics for the month of July.
- The right boundary condition was set to convection with $h_{room}=$5 W/(m^2^·K) and a constant temperature of 22 °C.

**Table S7.** Assumed numerical parameters, apparent density, and thermal conductivity of compositions.

| Parameter | Assumption | Parameter | Assumption |
| --- | --- | --- | --- |
| Wall thickness | 20 cm | *ρ* (CEM) | 2030 kg/m^3^ |
| Timestep | 300 s | *ρ* (CCS) | 1600 kg/m^3^ |
| Solar absorptance | 0.65 | *ρ* (CCPMF) | 1690 kg/m^3^ |
| Surface emissivity | 0.87 | *ρ* (CCPSiO_2_) | 1700 kg/m^3^ |
| Stefan-Boltzmann constant | 5.6704 × 10^-8^ kg/(s^3^.K^4^) | *k* (CEM) | 1.535 W/m.K |
| Room temperature | 22 °C | *k* (CCS) | 0.754 W/m.K |
| $\boldsymbol{h}_{\boldsymbol{room}}$ | 5 W/(m^2^·K) | *k* (CCPMF) | 1.069 W/m.K |
| $\boldsymbol{h}_{\boldsymbol{out}}$ | 25 W/(m^2^·K) | *k* (CCPSiO_2_) | 1.109 W/m.K |
